# Prevalence and Anatomical Characteristics of Bifid and Trifid Mandibular Canals: A Computer Tomography Analysis

**DOI:** 10.3290/j.ohpd.b5573959

**Published:** 2024-07-19

**Authors:** Alessandro Cuozzo, Iorio-Siciliano Vincenzo, Marius Boariu, Darian Rusu, Stefan-Ioan Stratul, Luigi Galasso, Vitolante Pezzella, Luca Ramaglia

**Affiliations:** a PhD Student, Department of Periodontology, University of Naples Federico II, Naples, Italy. Performed statistical evaluation, wrote the manuscript.; b Professor, Department of Periodontology, University of Naples Federico II, Naples, Italy. Contributed substantially to discussion the paper, proofread the manuscript.; c Associate Professor, Department of Endodontics, Faculty of Dental Medicine, TADERP Research Center, “Victor Babes” University of Medicine and Pharmacy, Timisoara, Romania. Edited the manuscript.; d Professor, Department of Periodontology, Faculty of Dental Medicine, Anton Sculean Research Center for Periodontal and Peri-Implant Diseases, “Victor Babes” University of Medicine and Pharmacy,Timisoara, Romania. Edited the manuscript.; d Dentist, Department of Periodontology, Faculty of Dental Medicine, Anton Sculean Research Center for Periodontal and Peri-Implant Diseases, “Victor Babes” University of Medicine and Pharmacy, Timisoara, Romania. Acquisition, analysis and interpretation of the data, wrote the manuscript.; e Oral Surgeon, Department of Periodontology, University of Naples Federico II, Naples, Italy. Acquisition, analysis and interpretation of the data, wrote the manuscript.; f Professor, Department of Periodontology, University of Naples Federico II, Naples, Italy. Study conception and design, contributed substantially to discussion the paper.

**Keywords:** anatomical variations, cone-beam computed tomography, mandibular canal, mandibular nerve, oral surgery, third molar

## Abstract

**Purpose:**

To assess the prevalence and configuration of bifid (BMC) and trifid (TMC) mandibular canals using computed tomography (CT), describing the anatomical characteristics of the accessory canals, especially of the retromolar type.

**Materials and Methods:**

CT scans of 123 patients were analysed. BMCs were identified and the patterns of bifurcation were classified, including trifid canals. The width of accessory canals was measured. Retromolar canals were further classified according to their course and morphology, while their position and width were evaluated using linear measurements on CT images.

**Results:**

The majority of patients (53.6%) presented at least one BMC or TMC. 36.2% of mandibular canals were bifid, while 4.5% were trifid. The forward canals (12.6%) and retromolar canals (10.2%) were the most common among BMCs. In relation to the retromolar canals, 60% were vertical and 40% curved, with a mean width of 1.03 ± 0.28 mm.

**Conclusion:**

BMCs and TMCs are common 3D radiographic findings, so that they should be considered as anatomical variations, not anomalies. Preoperative CT or CBCT evaluation should aid in identifying these variations and analysing their position and course in surgical planning.

The mandibular canal (MC) is an anatomical structure of great clinical importance since it is an intraosseous canal containing different structures which form the inferior alveolar bundle.^19^Determining the position and morphology of the mandibular canal could avoid damaging its neurovascular plexus during oral surgery. It is also advisable to identify its anatomical variations, such as bifid mandibular canals (BMCs) and trifid mandibular canals (TMCs).^26^ Indeed, the mandibular canal can present several ramifications, dividing it into a main branch, which keep its path to the mental foramen, and one or more accessory branches, which have a different course in the mandible.^1^

Several studies showed that accessory canals may contain neurovascular bundle.^4^^,^^7^^,^^12^ A lesion of these structures during oral surgery can lead to complications such as paresthesia, bleeding or traumatic neuroma.^5^

The accessory canals can originate in more or less posterior areas of the mandible and can run above, below or laterally (lateral or medial position) to the mandibular canal. They can describe different paths in the jaw and, eventually, they can present a confluence with the mandibular canal in a position anterior to the branching point.^14^

These accessory canals can be close to the alveolar process of the mandible and dental elements.^2^ A systematic review found that, in rare cases, an accessory canal can be entrapped between the roots of mandibular third molar.^11^

Moreover, neurovascular anatomy in retromolar region can be particularly complex: the main neural supply to the posterior mandible is by the inferior alveolar nerve but, occasionally, additional innervation is provided by branches from the lingual and mylohyoid nerves that may enter the posterior body of the mandible through lingual foramina.^3^

A cadaver study found that lingual branches of the mandibular canal have an intimate relationship with the lingual nerve and establish several anastomoses with other arteries on the poserior lingual aspect of the mandible. For this reason, the accessory canals can influence the success of ridge augmentation techniques applied in the posterior sector of the mandible.^23^

One of the most frequent accessory canals is the retromolar canal, a type of BMC which is often associated with surgical complications; this anatomical structure arises from the mandibular canal behind the third molar and travels anterosuperiorly to the retromolar foramen, located in the retromolar fossa.^28^

A recent systematic review found that the mean length of BMC was 12.38 ± 2.92 mm and the mean diameter of the BMC was 1.64 ± 0.46 mm.^21^

Different classification systems have been proposed for BMCs, based on the anatomical position and configuration of the accessory canals. The earliest classification systems, such as those of Nortje et al^16^^,^^17^ and that of Langlais et al,^10^ were based on panoramic radiographs, while Naitoh et al^14^ proposed a system based on cone-beam computed tomography (CBCT) images.

Localisation of accessory canals on panoramic radiographs is often difficult or impossible, due to the overlapping of buccal and oral bone structures and to the dimensional distortion due to the irregular magnification.^14^ Instead, computed tomography (CT) and CBCT provide three-dimensional images and allow an easier identification of position and width of accessory canals.^6^ However, there are no classifications including the trifid canal type, although many cases have been reported in previous publications.^20^

Hence, the aims of the study were to determine the prevalence and configuration of BMCs and TMCs using CBCT, and to describe in detail the anatomical characteristics (three-dimensional position, morphology and width) of the retromolar canal.

## MATERIALS AND METHOD 

The present study was conducted in accordance with the principles of the Declaration of Helsinki and the research protocol was approved by the Ethics Committee of the University of Naples “Federico II” (No. 286/20). Each patient signed a detailed informed consent form for processing personal data.

A selection of CT and CBCT examinations of patients treated at the Department of Oral Surgery, University of Naples Federico II from May 2018 to January 2020 were retrospectively collected and evaluated. The inclusion criteria were: males and females age ≥18 years; CT or CBCT performed for odontostomatological examinations (e.g., tooth extraction, dental implant planning); scans with a field of view (FOV) showing the full mandibular arch.

The exclusion criteria were: scans that did not clearly show the entire path of the mandibular canal (from mandibular foramen to mental foramen); scans with osteolytic and/or osteosclerotic lesions in the posterior area of the mandible dislocating or infiltrating the neurovascular bundle.

The majority of CT and CBCT examinations were requested for planning surgical extraction of one or both mandibular third molars, when panoramic radiographs showed an intimate relationship between dental roots and mandibular canal. A total of 138 scans were available for the analysis, while 15 scans were excluded. Ten scans did not show the entire path of the mandibular canal (in 6 scans, the mandibular branches had been excluded from image acquisition, while 4 scans had insufficient resolution to analyse in detail the course of any accessory canals). In addition, 5 scans showed the presence of an osteolytic lesion in the posterior region of the mandible.

The process of collecting CT and CBCT scans resulted in the selection of 123 patients (246 mandibular canals), of whom 64 (52%) were females and 59 (48%) were males, with a mean age of 31.4 ± 11.62 years and age range between 18 and 78 years.

Analysis of the tomographic scans and data collection were performed by two clinicians (V.P. and L.G.). Interexaminer agreement was analysed using the kappa (κ) coefficient. A κ-score of 0.90 was accepted as agreement. Any discrepancy between the two clinicians was resolved by discussion.

### Evaluation and Classification of Accessory Canals using CBCT Images

RadiAnt DICOM Viewer software (version 4.6.9; Medixant; Poznan, Poland) was used to process the projection data of the scans. For each evaluation site, the entire path of the mandibular canal was evaluated on multiplanar reconstruction (MPR) images: first, the clinician directed the sagittal reference line, on the axial plane, to pass through the retromolar fossa and the first mandibular premolar; and, on the coronal plane and in correspondence of the third molar region, to be pass through the alveolar ridge and the lower border of the mandible. Then, the clinician directed the axial reference line, on the sagittal plane, to be parallel to the lower border of the mandible. From these settings, the clinician could freely change the orientation of the reference lines and browse in the three planes to better study the anatomy and analyse any accessory canals. When necessary, brightness and contrast of the images were adjusted to optimise recognition and determination of the mandibular canal and any accessory canals.

For each evaluation site, the mandibular canal was classified as normal, bifid or trifid, according to the number of accessory canals branching from it (none, one or two).

The BMCs were classified according to the classification of Naitoh et al:^14^

- Retromolar canal (type I): branches off the mandibular canal in ramus region, runs anterosuperiorly and opens at the retromolar foramen in the retromolar region;
- Dental canal (type II): bifurcates from the mandibular canal and reaches the root apex of the second or third molar;
- Forward canal (type III): arises from the superior wall of the mandibular canal and runs forward parallel to the main canal, with or without confluence;
- Buccolingual canal (type IV): arises from the buccal or lingual wall of the mandibular canal.

The width of the accessory canals was measured immediately anterior to the bifurcation point on the coronal CT or CBCT images.

Retromolar canals were further classified into three categories based on their course and morphology, according to the classification of von Arx et al:^28^

Vertical (type A): arises from the mandibular canal and runs vertically upwards, with a more or less straight and direct course, up to the retromolar foramen;
Curved (type B): arises from the mandibular canal, runs in an anterior direction and then curves superoposteriorly towards the retromolar fossa;
Horizontal (type C): arises from the mandibular foramen and runs anteriorly in a horizontal direction to open on the anterior face of the mandibular branch or into the retromolar fossa.

Furthermore, sagittal CT- or CBCT-derived images were used to determine position and width of retromolar canals:^25^

Horizontal distance from the midpoint of the retromolar foramen to second molar (on the distal CEJ);
Height of retromolar canal: vertical distance from the midpoint of the retromolar foramen to the upper border of the mandibular canal;
Width of retromolar canal: measured at a level of 3 mm below the mesial aspect of the retromolar foramen.

In addition, we measured two other distances on the sagittal CT or CBCT images: distance from the mandibular foramen to the branch point of the retromolar canal; width of mandibular canal from which a retromolar canal branches off: measured immediately before bifurcation.

Scans were subjected to a second analysis using Simplant pro 18 software (version 18.0.0; Dentsply Implants NV; Hasselt, Belgium). This software was used to process the volumetric reconstructions and to visualise the mandibular canal and any accessory canals in three-dimensional space after having reconstructed them starting from axial, trans-axial and panoramic-like images.

The 3D rendering of the mandible allows rotating the reconstruction without constraints for a more accurate evaluation of the position and direction of the accessory canal and for a correct identification of any retromolar foramen.

### Statistical Analysis

Descriptive and comparative statistical analysis were performed by means of computer software (Microsoft Excel, Microsoft; Redmond, WA, USA and IBM SPSS; Chicago, IL, USA). The data were presented as frequencies, means and standard deviations. Differences in the prevalence rate of accessory canals according to gender, side and type were evaluated using the chi-squared test and Fisher’s exact test. ANOVA was used to evaluate differences in width among accessory canal types. A value of p < 0.05 was considered statistically significant.

## RESULTS

**Fig 1:**
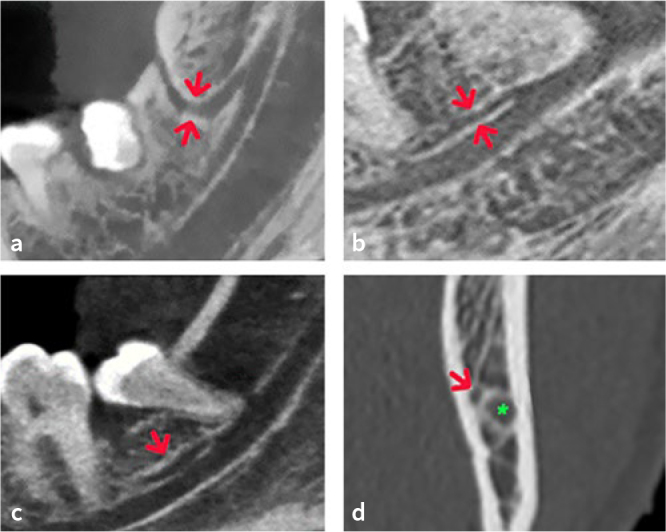
Multiplanar reconstruction CBCT images showing different types of BMCs: a. retromolar canal (red arrows); b. dental canal (red arrows); c. forward canal (red arrow); d. buccolingual canal (red arrow). Green asterisk indicates the mandibular canal.

A total of 53.6% patients (n = 66/123) had at least one bifid (Fig 1) or trifid mandibular canal, with a prevalence in female patients of 46.8% (n = 30/64) and of 61% (n = 36/59) in male patients (Table 1). Six scans did not show the initial course of the mandibular canal starting from the mandibular foramen (the mandibular branches were excluded from image acquisition, cutting the mandibular foramen and the initial course of the mandibular canal, bilaterally), 4 scans had insufficient resolution to analyse in detail the course of any accessory canals and 5 scans showed the presence of an osteolytic lesion in the posterior region of the mandible.

**Table 1:** Frequencies of accessory canals by gender

Gender	Accessory canals		Total	p-value*
	Present	Absent		
Male	36 (61%)	23 (39%)	59 (48%)	
Female	30 (47%)	34 (53%)	64 (52%)	0.148
Total	66 (53.6%)	57 (46.4%)	123 (100%)	

*p-value refers to the Fisher’s exact test.

No statistically significant differences in the prevalence of accessory canals for gender was found (p ≤ 0.05). In 66 patients with at least one bifid or trifid mandibular canal, 51.5% (n = 34/66) showed accessory canals bilaterally. In 27 patients, both mandibular canals were bifid, in 4 patients one canal was bifid and the contralateral one was trifid, and in only 3 patients were both canals trifid. The remaining 48.5% (n = 32/66) had one or two accessory canals only on the right or left side.

The prevalence of BMCs and TMCs was, respectively, 50.4% (n = 62/123) and 6.5% (n = 8/123). The prevalence on single sites (hemimandibles) was 36.2% (n = 89/246) for BMCs and 4.5% (n = 11/246) for TMCs. The most common variant among BMCs was the forward canal (type III;12.6%), followed by retromolar canal (type I; 10.2%), dental canal (type II; 6.2%) and buccolingual canal (type IV; 3.7%).

**Fig 2:**
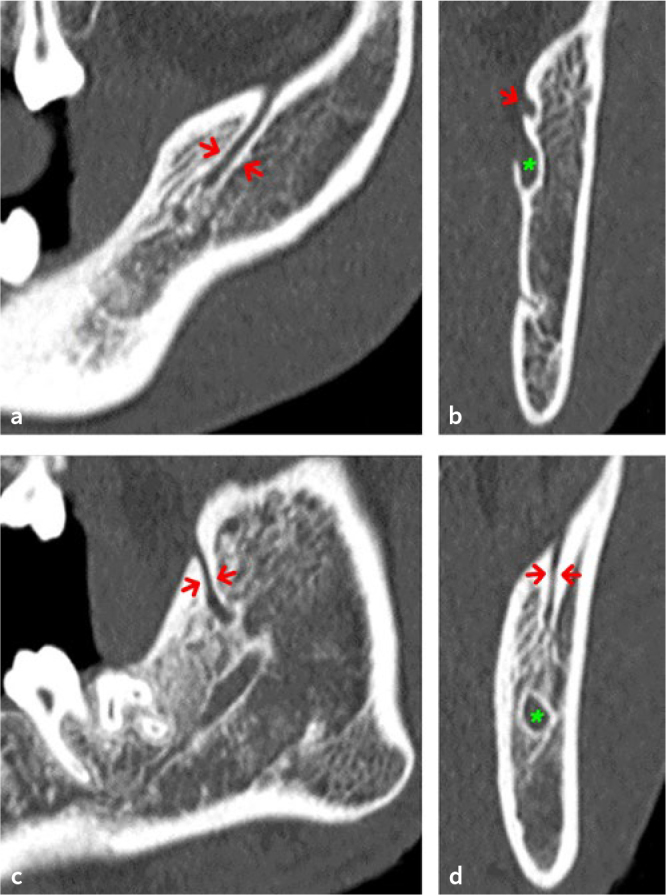
CBCT images of a temporal crest canal (TCC): a. Sagittal CBCT slice showing initial course of TCC (red arrows); b. coronal CBCT slice showing the mandibular foramen (green asterisk) and the accessory foramen (red arrow); c. sagittal CBCT slice showing the last part of TCC (red arrows); d. coronal CBCT slice showing the TCC opening at a bony foramen (red arrows) and the mandibular canal (green asterisk).

**Fig 3:**
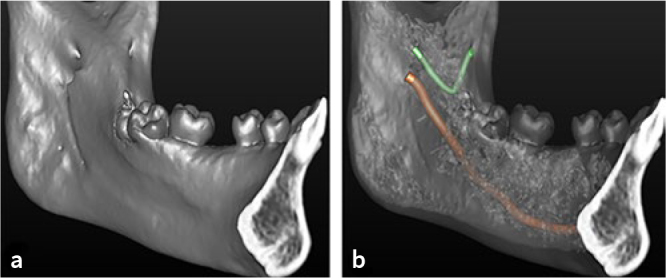
Temporal crest canal (TCC): a. Volumetric reconstruction showing the accessory foramen, positioned anteriorly and cranially to the mandibular foramen, and the exit foramen of the TCC; b. volumetric reconstruction with a transparency kernel that highlights the courses of the TCC and the mandibular canal.

The remaining 9 mandibular canals (3.7%; n = 9/246) were not classified, due to a path not included in the adopted classification: in eight evaluation sites, an accessory branch departing from the inferior wall of mandibular canal and running antero-inferiorly, while in one hemimandible a temporal crest canal (TCC) was observed (Figs 2 and 3).

No statistically significant differences in the prevalence of types of accessory canals were found depending on gender (p = 0.145) and side (right or left) (p = 0.742).

The prevalence and the mean width ± SD of diverse types of accessory canals were reported in Table 2. The mean width of retromolar canals (1.44 ± 0.65 mm) was statistically significantly greater than anterior canals (1.08 ± 0.26 mm; p = 0.043). Furthermore, no statistically significant differences were observed in the width of the other types of accessory canal.

**Table 2:** Prevalence, mean width and standard deviation of accessory canals

Canal type	n (%)	Mean width	SD	Range
Non-bifid canal	146 (59.3%)	-	-	-
Bifid mandibular canal (BMC) Retromolar canal (type I) Dental canal (type II) Forward canal (type III) Buccolingual canal (type IV) Not classifiable BMC	89 (36.2%) 25 (10.2%) 15 (6%) 31 (12.6%) 9 (3.7%) 9 (3.7%)	1.22 mm 1.44 mm* 1.27 mm 1.08 mm* 1.19 mm 1.00 mm	± 0.45 mm ± 0.65 mm ± 0.38 mm ± 0.26 mm ± 0.40 mm ± 0.28 mm	0.56 – 3.01 mm 0.66 – 3.01 mm 0.63 – 2.01 mm 0.57 – 1.49 mm 0.65 – 1.82 mm 0.56 – 1.48 mm
Trifid mandibular canal (TMC)	11 (4.5%)	1.18 mm	± 0.35 mm	0.62 – 1.80 mm
Total	246 (100%)	1.21 mm	± 0.44 mm	– 3.01 mm

*Statistically significant (p <0.05).

**Fig 4:**
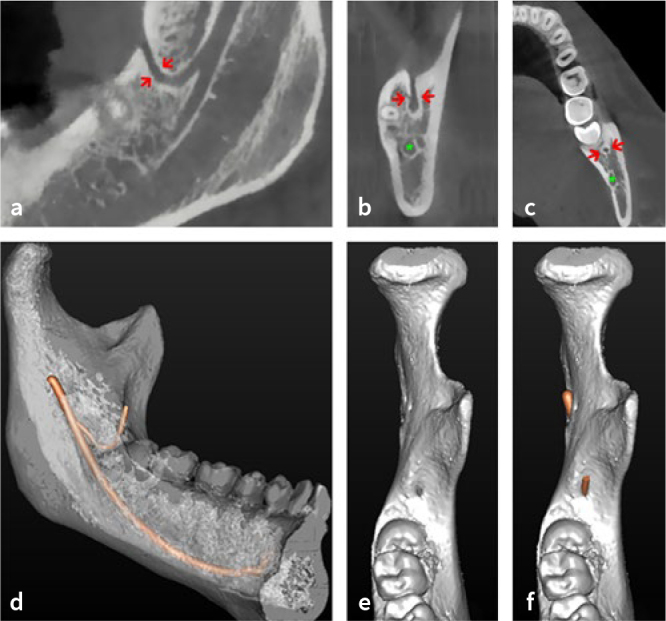
CBCT images of a retromolar canal: a. Sagittal CBCT slice showing a vertical retromolar canal (red arrows) from the bifurcation point to the retromolar foramen; b. coronal CBCT slice showing the last section of the retromolar canal (red arrows) and its opening (retromolar foramen) and the mandibular canal (green asterisk); c. axial CBCT slice showing a section of retromolar canal (red arrows) and a section of the mandibular canal (green asterisk); d. volumetric reconstruction with a transparency kernel that highlights the entire course of the retromolar canal; e. detail of the volumetric reconstruction showing the retromolar foramen; f. detail of the volumetric reconstruction showing the vascular-nerve bundle that crosses the retromolar foramen.

**Fig 5:**
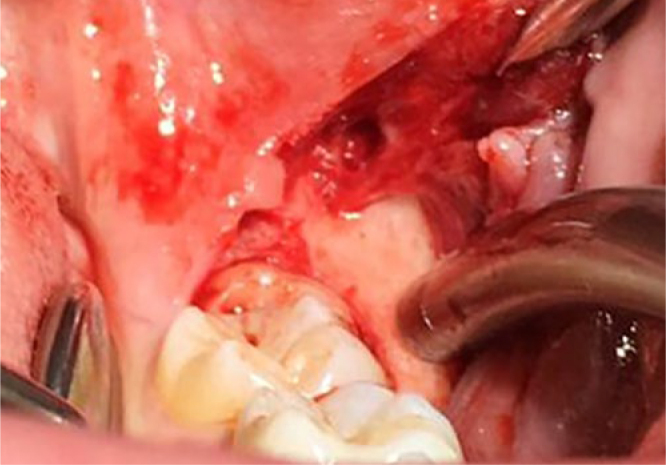
Flap dissection during the surgical extraction of tooth 38 shows the retromolar foramen.

A percentage of 15.5% of patients (n = 19/123) showed at least one retromolar canal and, of these, six patients presented it on both sides. Therefore, a total of 25 retromolar canals (10.2%) were detected with CBCT images at 246 sites (Figs 4 and 5). In relation to their morphology, 60% had a vertical pattern (n = 15/25, type A), while 40% had a curved course (n = 10/25, type B); the horizontal pattern (type C) was not seen.

The data related to the main position and width of the retromolar canals are summarized in Table 3. The distance from the midpoint of the retromolar foramen to the second molar was 15.7 ± 3.5 mm (range 11-29.3 mm). The height of the retromolar canal was 12.9 ± 2.7 mm (range 9.6-21.5 mm). The width was 1.03 ± 0.28 mm (range 0.45-1.47 mm). The distance from the mandibular foramen to the bifurcation point was 12.18 ± 5.32 mm (range 2.7 23.3 mm). The width of the mandibular canal from which the retromolar canals branch off was 3.12 ± 0.67 mm (range 1.96-4.44 mm).

**Table 3:** Linear measurements of the retromolar canals

Distance	Mean	SD	Range
Horizontal distance from midpoint of retromolar foramen to second molar	15.7 mm	3.5 mm	11 – 29.3 mm
Height of retromolar canal	12.9 mm	2.7 mm	9.6 – 21.5 mm
Width of retromolar canal	1.03 mm	0.28 mm	0.45 – 1.47 mm
Distance from the mandibular foramen to the branch point of the retromolar canal	12.18 mm	5.32 mm	2.7 – 23.3 mm
Width of mandibular canal from which the retromolar canal branches off	3.12 mm	0.67 mm	1.96 – 4.44 mm

## DISCUSSION

The present retrospective observational study analysed the prevalence and morphological characteristics of bifid and trifid mandibular canals using CT and CBCT. The present investigation explored the characteristics of the retromolar canals, establishing not only their diameter and position, but also analysing their relationships with the mandibular canal from which they branch (branching point and width of the mandibular canal). These outcomes should be considered to prevent possible surgical complications. A high prevalence of these anatomical variants was found (53.6%). The most frequent types of accessory canal were the forward canal (12.6%) and retromolar canal (10.2%); 3.7% of the accessory canals presented a course that is not described in the adopted classification. The mean width of accessory canals was 1.21 ± 0.44 mm. Retromolar canals were significantly wider than the other types: the mean width was 1.03 ± 0.28 mm, 60% were vertical and 40% curved. In the present study, the scans showing osteolytic and/or osteosclerotic lesions in the posterior area of the mandible dislocating or infiltrating the neurovascular bundle were excluded to avoid a modification of the original anatomy.

Previous studies analysed the accessory canals of the mandibular canal, considering CT or CBCT a suitable modality for a detailed evaluation.^15^^,^^18^^,^^24^ In other words, tomographic scans provide high-resolution three-dimensional images and they can detect narrow accessory canals and those that bifurcate in the buccal or lingual directions.^30^

To correctly evaluate the canals that branch out in a more posterior position of the mandibular canal, near the mandibular foramen, only tomographic scans which allowed an analysis of the whole mandible were included. Mandibles with osteolytic or osteosclerotic lesions in the posterior region were excluded because the pathology could have modified the original anatomy.

Finally, the retromolar canal (type I) has been subjected to a more accurate analysis since it represents one of the most frequent types of bifurcation and is more frequently associated with surgical complications.^25^ In agreement with previous studies (i.e., prevalence rate of BMCs ranging from 15.6% to 66.5%),^9^^,^^13^^,^^22^ the prevalence of BMCs was 50.4%. However, this outcome is in contrast to those reported by Naitoh et al^14^ and Orhan et al.^18^

In the present investigation, the prevalence of trifid mandibular canals (TMCs) was 6.5%. These results agreement with those reported in a previous study which reported a prevalence of 5.8%.^17^

The classification proposed by Naitoh et al^14^ was adopted to analyse the anatomical variants of BMCs. Forward (type III) and retromolar (type I) canals were the most frequent types recorded, with percentages of 34.83% and 28.09%, respectively. These data are confirmed by Naitoh et al^14^ and Orhan et al,^18^ who found a higher frequency of type III and I canals with a prevalence of 59.6% and 29.8% for forward canals, respectively, and of 29.8% and 28.1% for retromolar canals, respectively. The prevalence of dental (type II; 16.85%) and buccolingual (type IV; 10.11%) canals was in present study lower than type I and III; by comparison, Naitoh et al^14^ reported a rate of 8.8% for type II and 1.8% for type IV.

In addition, we also found some anatomical variation (3.7%; n = 9/246) not described by the classification used here.^14^ In eight hemimandibles, the accessory canal originated from the inferior wall of mandibular canal and running antero-inferiorly. Moreover, a rare anatomical variation, TCC, was found in one patient. It originated from an accessory mandibular foramen, positioned anteriorly and cranially to the mandibular foramen, ran first antero-inferiorly and then anterosuperiorly and opened at a bony foramen located in the anterior region of the temporal crest.^29^ Probably, TCC conveys the long buccal nerve and the associated blood vessels, pierces the temporalis tendon and travels to the cheek and mandibular buccal gingiva.^8^^,^^29^ The data on width and position of the retromolar canals of our study are in agreement with the results reported by von Arx et al,^28^ who applied the same measurement modalities, detecting a horizontal distance from midpoint of retromolar foramen to second molar of 15.2 ± 2.4 mm, a height of retromolar canal of 11.4 ± 2.7 mm and a width of retromolar canal of 1 ± 0.31 mm.

In the present investigation, a total of 123 tomographic exams were retrospectively collected and analysed. To draw conclusions supported by a higher level of evidence, a further study with larger sample may be needed.

Since the majority of the patients were referred from other dental centers, the CTs or CBCTs were already available. In order to reduce x-ray exposure, new radiographic exams were not proposed, which is a limitation of the study.

## CONCLUSION

A high prevalence of BMCs and TMCS was recorded, in particular anterior and retromolar canals. In addition, the retromolar canals were located distally to third molars with a greater diameter than other canals. These results are relevant for surgical treatment planning to decrease the risk of surgical and post-operative complications. For these reasons, an accurate CT or CBCT analysis is recommended prior to performing surgery.
